# The Influence of *Giardia duodenalis* on the Occurrence of Clinical Signs in Dogs

**DOI:** 10.3390/vetsci10120694

**Published:** 2023-12-07

**Authors:** Iva Šmit, Dalibor Potočnjak, Vesna Matijatko, Marin Torti, Ines Jović, Darko Grden, Martina Crnogaj, Relja Beck

**Affiliations:** 1 Clinic for Internal Diseases of the Faculty of Veterinary Medicine, University of Zagreb, 10000 Zagreb, Croatia; ismit@vef.unizg.hr (I.Š.);; 2 Department for Bacteriology and Parasitology, Croatian Veterinary Institute, 10000 Zagreb, Croatia

**Keywords:** *G. duodenalis*, dog, assemblage, gastrointestinal clinical signs

## Abstract

**Simple Summary:**

The role of *G. duodenalis* in the onset of a broad variety of clinical signs, from asymptomatic to acute and chronic diarrhea, is still being questioned. The aim of this study was to investigate the correlation between the presence of *Giardia duodenalis* and different *Giardia* assemblages detected in symptomatic and asymptomatic dogs and the occurrence of certain clinical signs. In fecal analysis, *G. duodenalis* and its assemblages and other parasites/pathogens were correlated with clinical signs in eighty-two dogs. Of all the dogs, 42 had gastrointestinal clinical signs and *G. duodenalis* was found in 30.5% of dogs, 40% of which presented with assemblage C and 60% with assemblage D. *G. duodenalis* was more common in shelter dogs. Of other parasites, only *Cryptosporidium* spp. showed a higher coinfection rate with *G. duodenalis* but that did not have an influence on clinical sign appearance. There was no correlation between the presence of different assemblages of *G. duodenalis* and the sex of the host or the duration and appearance of certain clinical signs, except the presence of mucus in feces, which was more frequent in dogs invaded with *G. duodenalis* assemblage C.

**Abstract:**

*Giardia duodenalis* infections are common in dogs and are mainly caused by assemblages C and D. The aim of this study was to investigate the correlation between the presence of *Giardia duodenalis* and different *Giardia* assemblages detected in symptomatic and asymptomatic dogs and the occurrence of certain clinical signs. All the dogs included (*n* = 82) were clinically examined, and fecal samples were examined for other parasites and *Clostridium* spp. Also, *G. duodenalis* assemblages were detected and the occurrence of certain clinical signs was assessed. A total of 42/82 (51.2%) dogs were symptomatic and had one or more gastrointestinal signs, and 40/82 (48.8%) dogs were asymptomatic. *G. duodenalis* was found in 25/82 (30.5%) dogs: assemblage C in 10/25 (40%) and assemblage D in 15/25 (60%). Only *Cryptosporidium* spp. showed a higher coinfection rate with *G. duodenalis* but that did not have an influence on clinical sign appearance. There was no correlation between the presence of different assemblages of *G. duodenalis* and the sex of the host or the duration and appearance of certain clinical signs, except the presence of mucus in feces, which was more frequent in dogs invaded with *G. duodenalis* assemblage C. Further research of other assemblages is needed.

## 1. Introduction

*Giardia* spp. are diplomonad flagellates found in a broad range of vertebrates. Among them, *Giardia duodenalis* (*G. duodenalis*) is now known as a multispecies complex of highly specific assemblages. Assemblages C, D, E, F, G, and H have been detected in various species of domestic and wild animals, while assemblages A and B have mainly been isolated from humans but also from various species of domestic and wild animals. *G. duodenalis* parasites have a direct lifecycle with two stages: the proliferating trophozoite and infectious cyst. Parasites are transmitted to a host when infectious cysts are ingested via contaminated water or food or by direct fecal–oral contact. On exposure to the environment of the gastrointestinal tract of a host, infective cysts begin the process of excystation which results in the release of trophozoites. Trophozoites are the disease-causing stage as they attach to enterocytes in the upper small intestine of the host. Here, they divide and absorb nutrients from their host. They transform into infective cysts [1,2,3,4]. Over the last thirty years, most studies on giardiasis in dogs have concentrated on understanding its prevalence, genetic diversity, and zoonotic potential. *G. duodenalis* is the most prevalent parasite of dogs worldwide [5,6,7,8,9]. Its prevalence varies from 5 to 100% in privately owned dogs and depends on different factors such as age, diagnostic test, health status, gut microbiota, geographical region, veterinary visit time, etc. [10,11,12,13]. The prevalence of gastrointestinal parasites in shelter dogs is typically higher than in privately owned dogs, probably due to large concentrations of animals, arrivals of new dogs (including puppies, which are at a great risk of parasite infection), hygiene, and facility management. Shelters provide a perfect environment that could facilitate the spread of parasitic infections between large numbers of animals [13].

The role of *G. duodenalis* in the onset of a broad variety of clinical signs, from asymptomatic to acute/chronic diarrhea, is still being questioned. Clinical signs in infected dogs are inconsistent and the role of *G. duodenalis* in other gastrointestinal diseases is still unknown. In a research study conducted in Australia, it was shown that *G. duodenalis* could be responsible for acute or chronic diarrhea cases or chronic waxing and waning gastrointestinal signs in dogs [14]. Furthermore, its prevalence in dogs with diarrhea was significantly higher among client-owned dogs than among stray dogs [15], but the prevalence of *G. duodenalis* in asymptomatic and symptomatic dogs was almost identical. The disease range can vary from asymptomatic to manifested clinical signs, depending on the age and nutritional status of the animals, as well as other factors and comorbidities. The most frequent clinical sign of giardiasis is diarrhea, which can be acute or chronic, self-limiting, intermittent, or continuous and lead to dehydration and it can differ in severity and frequency. As a result of the their inflammatory reaction, infected animals may develop severe enteritis, resulting in abdominal pain, nausea, maldigestion, and malabsorption. Some dogs may experience foul-smelling diarrhea, steatorrhea, weight loss, and decreased growth. The majority of infected immune-competent dogs represent carriers without displaying obvious clinical signs and can be a source of infection for other animals and humans [16]. Host factors that influence the severity of disease are likely to relate to age, immune response, nutritional status, coinfections with other gastrointestinal pathogens, and microbiome composition [17]. Several studies [11,18,19,20] concluded that *G. duodenalis* infection affects the intestinal microbiota of dogs, potentially leading to dysbiosis-related diseases. Changes in the intestinal microbiome by other enteropathogens are commonly associated with gastrointestinal diseases and have important consequences for overall health. *G. duodenalis* infection can cause altered intestinal microbiota species composition, functional changes in commensal microbiota, and changes to intestinal bacterial biofilm structure and these are the factors that may contribute to a number of acute or chronic clinical manifestations [19]. Besides common gastrointestinal signs, some dogs show other, uncommon clinical signs, such as cutaneous lesions and urticaria [21].

Some studies suggest a possible relation between *G. duodenalis* assemblage and the severity of clinical disease. One study in dogs reported that dogs with diarrhea most likely harbored dog-specific assemblages C and D [22]. Other studies failed to associate diarrhea with *G. duodenalis* assemblages or other parasitic coinfection [23,24]. Perrucci et al. (2020) [25] found significantly less severe clinical forms in *G. duodenalis*-positive dogs with chronic enteropathy than in noninfected dogs. Coinfections with different pathogens, parasites, bacteria, or viruses are also a possible predisposing factor in the development of gastrointestinal clinical signs [26] and in inducing changes in the microbiome, which can also contribute to gastrointestinal clinical signs [27]. The parasites that commonly cause gastrointestinal clinical signs in dogs are *Giardia duodenalis*, *Ancylostoma caninum, Isospora canis, Uncinaria stenocephala*, and *Trichuris vulpis,* and their concurrent invasion is also very common [28]. *Clostridium perfringens* is a widespread, spore-forming Gram-positive anaerobic bacillus that inhabits the gastrointestinal tract of animals and humans. Based on the possession of one or more of four major toxin genes, it is divided into five different biotypes. The pathogenesis of *C. perfringens*-associated diarrhea in dogs is not fully understood because *C. perfringens* has also been detected in some nondiarrheic dogs [29].

We aimed to investigate the impact of *G. duodenalis* infection on the development of gastrointestinal clinical signs in dogs. Furthermore, we aimed to determine whether there is a correlation between infection with different *G. duodenalis* assemblages and the occurrence of gastrointestinal clinical signs or between coinfection with other pathogens and *G. duodenalis* and the occurrence of gastrointestinal clinical signs. This information could prove to be useful for better understanding of the pathogenesis of *G. duodenalis* infection in dogs.

## 2. Materials and Methods

### 2.1. Animals

In the current retrospective study, 82 client-owned (*n* = 58) and shelter dogs (*n* = 24) were included. The included animals were presented to the clinic for various reasons, including gastrointestinal clinical signs, routine, and general health examinations. Of those dogs, 42 had clinical signs and 40 were asymptomatic. The dogs included in our study were of both sexes, various breeds, adult (older than one year), and properly vaccinated. All the dogs included in this study were examined in the Clinic for Internal Diseases of the Faculty of Veterinary Medicine, University of Zagreb. Only adult dogs were included because puppies are at a greater risk of developing infectious diseases as their immune system is in development and structural changes in the gut microbiota are developed with age. To be included in the study, dogs had to have been vaccinated against distemper virus (CDV), canine adenovirus type 1 (CAV1), canine adenovirus type 2 (CAV2), canine parainfluenza virus (CPiV), canine parvovirus (CPV), and canine parvovirus type 2c (CPV2c). The exclusion criteria were the presence of an underlying gastrointestinal disorder, like inflammatory bowel disease, food intolerance, foreign body, or other gastrointestinal diseases.

### 2.2. Clinical Analysis

All the dogs were clinically examined; a detailed history was taken, blood samples for complete blood count and biochemistry analysis were taken, and fecal samples were collected. Every dog that had one or more clinical signs was considered symptomatic. Based on history, clinical examination, and laboratory tests, a list of 14 signs/indicators was established to evaluate the occurrence of gastrointestinal clinical signs. Clinical signs were selected by expanding the clinical sign list in the existing canine chronic enteropathy activity index [30] and they included the course of disease, decrease in physical activity, decreased appetite, vomiting, feces consistency, increase in defecation frequency (3 or more/d), hematemesis, fresh blood in feces, melena, fecal mucus, weight loss, decrease in serum albumin level (serum albumin ≤ 20 g/L), ascites and/or peripheral edema, and pruritus. The selected clinical signs were assessed as present or absent. Additional diagnostic tests were performed to exclude underlying disorders and to evaluate potential changes caused by *G. duodenalis* infection and included CBC and serum biochemical analysis, urinalysis, and imaging, if indicated. An acute course of disease was considered less than 3 weeks of duration and a chronic course of disease was considered more than 3 weeks of persistent or recurrent gastrointestinal clinical signs [31]. Stool consistency was estimated by using a fecal consistency scoring system. Normal feces consistency was firm, but not hard, pliable, segmented in appearance, with little or no residue on the ground when picked up. (https://www.purinainstitute.com/centresquare/nutritional-and-clinical-assessment/purina-fecal-scoring-chart), accessed on 1 August 2023.

### 2.3. Fecal Analysis

Dog fecal samples were collected immediately after defecation, stored in 50 mL sterile containers, and delivered within 8 h to the Parasitology and Microbiology Laboratory of the Croatian Veterinary Institute for parasitological and microbiological analysis. All 82 samples were analyzed with IFA Merifluor^®^ Cryptosporidium/Giardia (Meridian Bioscience, Luckenwalde, Germany) following the manufacturer’s instructions. This method was used to detect the presence of Giardia cysts and Cryptosporidium oocysts by visualization of fluorescein isothiocyanate (FITC)-conjugated antibodies. Three grams of feces was used for centrifugal flotation using magnesium sulfate (MgSO_4_; specific gravity 1.20) according to procedure described by Dryden et al. (2005) [32] for the detection of other parasites. *Giardia* cyst-positive samples were chosen for DNA extraction using the QIAamp^®^ DNA Stool Mini Kit (Qiagen, Hilden, Germany) following the manufacturer’s protocol. To increase the purity of the DNA, all extracted samples were further purified with the QIAquick^®^ PCR Purification Kit (Qiagen, Hilden, Germany). A nested PCR that amplified a portion of 175 small ribosomal subunit (SSU rRNA) loci was used for assemblage discrimination [33]. PCRs were performed in total volumes of 50 µL using 2 µL of DNA extracted in the first reaction and 5 µL of the PCR product from the first reaction in the nested PCR. The amplified products were analyzed by capillary electrophoresis (QIAxcel System^®^, QIAGEN, Hilden, Germany) with size markers in the range of 100–2500 bp. Samples were purified with ExoSAP-IT^®^ (USB Corp., Cleveland, OH, USA) and sequenced in both directions by Macrogen Inc. (Amsterdam, The Netherlands). Sequences were assembled using SeqMan Pro software 12.2.0 (DNASTAR, Madison, WI, USA), edited with EditSeq using Lasergene software 12.2 (DNASTAR, Madison, WI, USA), and compared with available sequences using BLAST. The isolation of the bacterium *Clostridium perfringens* was performed to identify it as a possible pathogen regardless of the possibility of toxin formation, but as a possible factor present in clinical sign development. After isolation and incubation, identification of the mentioned bacteria was carried out based on the morphological characteristics, a catalase and oxidase test, and a biochemical assay (BBL CrystalTMIdentification systems, 5.05A, Anaerobe ID kit) which served for the identification of anaerobic bacteria using conventional fluorogenic and chromogenic substances. An invasion score was determined and was defined as the total number of parasites/pathogens, and it was formed by summing all the types of parasites/pathogens found in a single sample.

### 2.4. Statistical Analysis

Data are presented as medians, minimums, or maximums, depending on the distribution of the data. Values were compared using the t-test or the test for nonparametric values (Mann–Whitney U test). The chi-square test or Fisher’s exact test were used to analyze data in binary form. Statistical significance was set at *p* < 0.05. All statistical analyses were performed using the commercially available software system Stata 13.1 (Stat Corp., College Station, TX, USA).

## 3. Results

There were 36 mixed-breed dogs and 46 pure-breed dogs (23 different dog breeds), ranging in age from 1 to 13 years (median 4 years old). The most common breeds were German Shepherd (*n* = 4), Poodle (*n* = 4), Belgian Shepherd (*n* = 3), Border Collie (*n* = 3), and Labrador Retriever (*n* = 3), and other breeds counted two or one dogs. The proportion of males and females was both 50%. *G. duodenalis* was present in 25/82 (30.5%) of all the dogs and an almost identical prevalence was found in both asymptomatic 12/40 (30.0%) and symptomatic dogs 13/42 (30.9%). *G. duodenalis* was found in 10/58 (17.2%) privately owned dogs and in 15/24 (62.5%) shelter dogs (*p* < 0.001).

To understand the role of coinfections, we analyzed the presence of other gastrointestinal parasites. *Giardia* was solely responsible for diarrhea in four symptomatic dogs (9.5%) and as coinfection in nine symptomatic dogs (21.4%). The incidence of intestinal parasites in all dogs was 43.9%. The most prevalent parasite, other than *G. duodenalis*, was *Cryptosporidium* spp. 10/82 (12.2%), followed by *Trichuris vulpis* in 8/82 (9.7%), *Toxocara canis* 6/82 (7.3%), *Isospora caninum* 4/82 (4.9%), *Strongyloides* spp. 2/82 (2.4%), and *Toxascaris leonine* 1/82 (1.2%). In *G. duodenalis*-positive dogs, there was no significant coinfection except with *Cryptosporidium* spp. Despite the significant coinfection rate of *G. duodenalis* with *Cryptosporidium* spp. (*p* < 0.0001), a correlation of coinfection with the presence of clinical signs was not confirmed (*p* = 0.376). The observed median invasion score between dogs with and without *G. duodenalis* was statistically different. Dogs with *G. duodenalis* were more often simultaneously infected with two or more parasites (*p* < 0.0001). Dogs with *G. duodenalis* had a median invasion score of 3, while dogs without *G. duodenalis* had a median invasion score of 1. *Clostridium perfringens* was detected in 70.7% of dogs (58/82) but was not involved in the development of clinical signs (*p* = 0.189).

In the group of dogs in which we had isolated *G. duodenalis*, 12 were female (48%) and 13 were male (52%) (*p* = 0.699).

There was a lack of correlation of *G. duodenalis* infection with the occurrence of clinical signs (Table 1). The prevalence of clinical signs in dogs with *G. duodenalis* was similar to that in dogs without *G. duodenalis*. Only the course of disease was different between these two groups of animals (*p* = 0.009). In dogs infected with *G. duodenalis*, the number of cases with a chronic course was higher than in noninfected dogs.

In the current study, dogs harbored only host-specific assemblages C and D. The obtained sequences were BLAST against Sprong et al. (2009) reference sequences [34] and found to be identical to reference sequences from assemblages C (AF199449) and D (AF199443). The current study’s sequences were deposited in GenBank under accession number SUB13938224 GDIS1 OR769666 andSUB13938244 GDIS2 OR769667. Assemblage D had a higher prevalence, 15/25 (60%), than assemblage C, 10/25 (40%). In privately owned dogs that tested positive for *G. duodenalis,* assemblage C was found in 4/10 dogs (40%) and assemblage D in 6/10 (60%) dogs, and in shelter dogs, assemblage C was found in 6/15 dogs (40%) and assemblage D in 9/15 (60%) dogs (*p* = 1.0). Almost identically, 50% of dogs infected with assemblage C and 53.3% of dogs infected with assemblage D were symptomatic. Furthermore, the proportions of female and male dogs were not different regarding *G. duodenalis* assemblage (*p* = 0.141). The duration of clinical signs was not attributable to a specific assemblage. In dogs infected with *G. duodenalis* assemblage C, the mean duration of symptoms was 19,4 days, and in dogs infected with assemblage D, it was 22,9 days (*p* = 0.739). The observed differences in invasion score between dogs with *G. duodenalis* assemblage C (median invasion score 2.5) compared to dogs with *G. duodenalis* assemblage D (median invasion score 3) were not significant (*p* = 0.50). There was no difference in the occurrence of most of the gastrointestinal and other clinical signs in dogs infected with assemblage C or D. Only fecal mucus was more prevalent in dogs infected with assemblage C (Table 2).

## 4. Discussion

Parasites are an important group of pathogens that can cause gastrointestinal infections. The prevalence of intestinal parasites in dogs varies between 16 and 72% [35,36,37,38,39], and the overall prevalence of 43.9% found in our study is within previously reported values. *G. duodenalis* is one of the most common gastrointestinal parasites in dogs worldwide [40]. This finding was also confirmed in the current investigation, which had an overall prevalence of *G. duodenalis* of 30.5%. One of the most significant public health concerns is the zoonotic potential of *G. duodenalis* isolated from dogs [41]. So far, it seems unlikely that it presents a risk for human infection in Croatia, as only host-adapted assemblages C and D have been confirmed in previous investigations in Croatia [42] and in this part of Europe [28,43,44,45]. The present study’s findings support previously mentioned results since the same assemblages were discovered, and none of these assemblages represent a potential zoonotic risk for humans. Highly specific assemblages are known to be more adapted to their hosts, replicate more quickly, and can displace assemblages A and B [46]. In contrast to these findings, multiple studies have discovered the possibly zoonotic assemblage A to be dominant (more than 80%) in dogs [22,47]; furthermore, assemblages A and B were less common, yet present in several studies [48,49]. Sequencing other genetic markers such as beta-giardin (BG), glutamate dehydrogenase (GDH), and triose phosphate isomerase (TPI) genes could provide more insight into the presence of subassemblages and subtypes, or in some cases mixed infections, as observed in previous research [50]. Additional studies on other markers have not been used here because links between the assemblages and the severity of clinical symptoms or even *G. duodenalis* infection were lacking. The present study’s finding of a significantly higher prevalence of *G. duodenalis* in shelter dogs than in privately owned dogs also supports the previously mentioned results and it was expected due to the large concentration of animals and easy spread of infections in shelters and similar facilities [13].

The role of *G. duodenalis* in the development of clinical signs is still poorly understood. Giardiasis in dogs can be a symptomatic or an asymptomatic disease [16]. Some studies have identified *Giardia* as a causal agent of diarrhea in dogs. The prevalence of *G. duodenalis* in dogs with diarrhea is 1.5- to 2-fold higher than in healthy dogs [15,35,40]. On the other hand, several studies showed a similar prevalence in asymptomatic and symptomatic dogs [23,25], as was the case in our study. In a study by Scorza et al. (2021) [24], diarrhea was not associated with any *Giardia* assemblage or other parasitic coinfection. Asymptomatic and symptomatic dogs had nearly identical *G. duodenalis* infection rates.

Diarrhea and vomiting are the most common clinical signs of digestive system disease [51]. The causes of diarrhea are diverse and numerous, requiring a wide range of medical diagnostic procedures and treatments. As a result, determining the source of diarrhea is crucial. *G. duodenalis, Ancylostoma caninum, Isospora canis, Uncinaria stenocephala,* and *Trichuris vulpis* are the parasites that most commonly cause gastrointestinal signs in dogs, either alone or in coinfections [14,36,37,39]. In the current study, *G. duodenalis* was involved in 30.3% of symptomatic dogs; it was the sole pathogen in 9.2% of symptomatic dogs, and in 21.4% of symptomatic dogs, other pathogens were isolated along with *G. duodenalis*.

*G. duodenalis* infection can have a wide range of clinical manifestations [16]. The most common symptoms in our study were altered feces consistency, increased frequency of defecation, mucus in feces, decreased appetite, decreased physical activity, and weight loss. These findings are in agreement with studies on the clinical presentation of giardiasis in dogs [52]. Several studies [24,25] showed that the presence of *G. duodenalis* was not related to severe clinical manifestations. In our study, the incidence of gastrointestinal clinical signs found in symptomatic dogs with *G. duodenalis* was nearly equal to that observed in dogs without *G. duodenalis,* but the disease course differed. Progression to chronic disease was predominant in *G. duodenalis*-infected dogs. Previous studies suggest that *G. duodenalis* is a prevalent cause of chronic diarrhea in dogs, but acute symptoms can also occur [16]. Interestingly, acute clinical presentation with systemic manifestation is more commonly recorded in infected human patients [53,54,55], whereas this condition is less common in animals [21].

Furthermore, despite the fact that dogs infected with *G. duodenalis* were more frequently coinfected with other parasites/pathogens than dogs not infected with *Giardia*, the degree of clinical alterations was unaffected. Tupler et al. (2012) [15], on the other hand, discovered that dogs with diarrhea were considerably more likely to be infected with more than one enteropathogens than dogs with normal feces. Because *Cryptosporidium* spp. and *G. duodenalis* share the same infection pathway, a high level of coinfection with *Cryptosporidium* spp. was to be expected. Both parasites are assigned to the group of water-borne parasites, so that the same sources of invasion can be assumed. These can be water puddles, wetlands in parks, or similar. These findings are consistent with prior research aimed at defining the most common concurrent infections of these parasites in dogs [56,57]. A higher invasion score in dogs infected with *G. duodenalis* is a logical consequence of the fact that *G. duodenalis* is more often found in dogs from shelters. Although it is unclear whether sex influences the prevalence of *G. duodenalis*, Upjohn et al. (2010) [58] and Meireles et al. (2008) [59] discovered a higher incidence of *G. duodenalis* infection in bitches. Pallant et al. (2015) found assemblage D to be more dominant in male than female dogs [60]. In the current investigation, there was no association between sex and assemblage prevalence. Other risk variables, such as animal housing, living routines, and even breed traits [61,62], appear to be more important than animal sex.

There has been little research into the relationship between *G. duodenalis* assemblages and clinical signs in dogs. Scorsa et al. (2021) [24] concluded that *Giardia* assemblages are not connected with diarrhea, while Uiterwijk et al. (2020) discovered that none of the assemblages were associated with loose feces [23]. One of the objectives of this study was to investigate a correlation between the *G. duodenalis* assemblages and the occurrence of various clinical signs in dogs, since such studies are sporadic in veterinary medicine. There was no difference in the occurrence of clinical signs among assemblages despite extensive investigation of 14 different clinical criteria, except for the presence of mucus in feces—this was seen in all dogs infected with assemblage C and in 37.5% of dogs infected with assemblage D. The presence of mucus in feces is primarily associated with pathological processes in the colon; however, given the poorly understood pathogenesis of giardiasis, it is believed that infections by these flagellates may favor the occurrence of other digestive tract diseases such as inflammatory bowel disease, disruption of physiological microflora, induction of intestinal motility disorders, and apoptosis of intestinal epithelial cells. These pathways cause intestinal glandular tissue hypersecretion and mucus production [12]. In general, it appears that diverse assemblages have no effect on the development of clinical signs. There is a lot of research conducted on the influence of assemblages A and B in the development of diarrhea in people, but the published results are somewhat conflicting. While several studies failed to show variations in clinical signs in human patients based on assemblages [63,64], others did [65,66,67]. Unfortunately, when clinical indications are assigned to assemblages A or B, the results in different research studies are contradictory [65,66,67].

Investigating the influence of *G. duodenalis* assemblages on the occurrence of digestive signs in dogs is a very challenging task as it requires taking into consideration multiple factors that can influence gastrointestinal clinical signs in dogs, such as other possible causes of diarrheal syndrome and intestinal malabsorption, like infections, food intolerances, endocrine disorders, etc. The existence of many factors that influence the appearance of clinical signs in dogs, as well as simultaneous infections with different pathogens, makes it very difficult to draw conclusions about the pathogenesis of the disease. The luminal microbial environment and its correlation with intestinal parasites is still an area that requires additional research.

The main limitations of this study include the small number of dogs enrolled, which may have prevented us from finding further differences between study groups, and the fact that no clinical condition follow-up was conducted.

## 5. Conclusions

We identified only canine-specific assemblages C or D in dog fecal samples. The presence of *G. duodenalis*, as well as different assemblages, had no effect on gastrointestinal clinical signs in dogs except the presence of mucus in feces. Finding equal rates of *G. duodenalis* infection in symptomatic and asymptomatic dogs is probably the consequence of many different factors such as host immune system, gut microbiota, parasite adaptation to the host, the fact that we found only assemblages C and D which are host-specific, etc. The presence of mucus in feces was seen in all dogs infected with assemblage C, compared to only 37.5% of dogs infected with assemblage D. This is a reason for further research on the pathogenesis of giardiasis and its influence on the immune system, gut microbiota, motility disorders, or other pathways that cause intestinal glandular tissue hypersecretion and mucus production. As a further step in research, the influence of infection by different assemblages on clinical signs and therapy duration or development of drug resistance should be studied.

## Figures and Tables

**Table 1:** Correlation of *G. duodenalis* infection with clinical signs.

Indicator/Clinical Sign		Giardia		*p*
		Negative	Positive	
Clinical course	Acute	23	5	0.009
	Chronic	6	8	
Decrease in physical activity	No	18	9	0.654
	Yes	11	4	
Decrease in appetite	No	17	9	0.513
	Yes	12	4	
Vomiting	No	19	11	0.205
	Yes	10	2	
Change in feces consistency	No	2	1	0.926
	Yes	27	12	
Increased defecation frequency	No	8	2	0.391
	Yes	21	11	
Weight loss	No	18	9	0.654
	Yes	11	4	
Decrease in serum albumin level	No	28	12	0.550
	Yes	1	1	
Pruritus	No	28	13	0.498
	Yes	1	0	
Ascites/edema	No	29	12	0.131
	Yes	0	1	
Hematemesis	No	28	13	0.498
	Yes	1	0	
Fecal mucus	No	16	5	0.317
	Yes	13	8	
Hematochezia	No	19	10	0.205
	Yes	11	2	
Melena	No	28	13	0.498
	Yes	1	0	

**Table 2:** Prevalence of clinical signs in groups of dogs with *G. duodenalis* assemblage C (*n* = 5) and assemblage D (*n* = 8).

Clinical Sign *		Assemblage C Positive Dogs (*n* = 5)	Assemblage D Positive Dogs (*n* = 8)	
Decrease in physical activity	%	40%	25%	*p* = 0.569
	N	2	2	
Decrease in appetite	%	20%	37.5%	*p* = 0.506
	N	1	3	
Vomiting	%	20%	12.5%	*p* = 0.715
	N	1	1	
Change in feces consistency	%	100%	87.5%	*p* = 0.411
	N	5	7	
Increased defecation frequency	%	80%	87.5%	*p* = 0.715
	N	4	7	
Weight loss	%	20%	37.5%	*p* = 0.506
	N	1	3	
Decrease in serum albumin level	%	0%	12.5%	*p* = 0.411
	N	0	1	
Ascites/edema	%	0%	12.5%	*p* = 0.411
	N	0	1	
Fecal mucus	%	100%	37.5%	*p* = 0.024
	N	5	3	
Hematochezia	%	40%	0%	*p* = 0.052
	N	2	0	
Chronic clinical course	%	40%	75%	*p* = 0.207
	N	2	6	

* Pruritus, hematemesis, and melena were not found in *G. duodenalis*-positive dogs.

## Data Availability

The data presented in this study are available on request from the corresponding author. The data are not publicly available due to owner privacy restrictions.
